# Static and Cyclic Mechanical Behavior of 3D-Printed PEEK Under Tensile and Compressive Loads

**DOI:** 10.3390/polym18060748

**Published:** 2026-03-19

**Authors:** Francisco Pina, Carlos M. S. Vicente, Joaquim Justino Netto, Luís Reis

**Affiliations:** 1 IST, Instituto Superior Técnico, Universidade de Lisboa, Av. Rovisco Pais, 1049-001 Lisbon, Portugal; franciscopina01@tecnico.ulisboa.pt (F.P.); joaquim.netto@tecnico.ulisboa.pt (J.J.N.); 2 Atlântica—Instituto Universitário, Fábrica de Pólvora de Barcarena, 2730-036 Barcarena, Portugal; carlos.vicente@tecnico.ulisboa.pt; 3 Center for Mechanical and Aerospace Science and Technologies (C-MAST-UBI), University da Beira Interior, R. Marquês de D’Ávila e Bolama, 6201-001 Covilhã, Portugal; 4 IDMEC, Instituto Superior Técnico, Universidade de Lisboa, Av. Rovisco Pais, 1049-001 Lisbon, Portugal

**Keywords:** compression fatigue, mechanical characterization, polyether ether ketone (PEEK), 3D printing, annealing

## Abstract

Polyether ether ketone (PEEK) is a high-performance polymer with exceptional mechanical properties, durability and lightweight. 3D printing of PEEK can be very beneficial in the medical industry to manufacture patient-specific implants; however, there is a lack of studies regarding the fatigue behavior of 3D-printed PEEK, especially under compression, which is closely related to its potential applications. This paper investigates the static and dynamic mechanical performance of 3D-printed PEEK. Tensile and compression tests were conducted on specimens with ±45° raster orientation. Annealing at 270 °C for 5 h increased crystallinity from 34.4% to 41.4% yet unexpectedly reduced tensile strength from 60.8 MPa to 47.3 MPa, while increasing Young’s modulus from 2.51 GPa to 3.51 GPa. Micro-CT analysis revealed increased pore size after annealing. Static compression strength showed improvement post-annealing, increasing from 80.1 MPa to 126.7 MPa, with modulus rising from 1.64 GPa to 2.28 GPa. Compression–compression fatigue tests, performed at 5 Hz and 2.5 Hz with stress amplitudes of 70–95% of maximum strength (R = 0.1), enabled the construction of the first S-N curve for 3D-printed PEEK under compressive loading. Annealed specimens exhibited superior fatigue life, with infinite life achieved at 83.3 MPa (70% of static strength). Thermal imaging highlighted the role of temperature in fatigue failure, showing that annealed specimens endured higher thermal loads. These findings support the suitability of 3D-printed PEEK for load-bearing biomedical applications under cyclic compressive loads.

## 1. Introduction

Additive manufacturing (AM), also known as three-dimensional (3D) printing, allows for the production of complex parts by adding material layer by layer until the final geometry is achieved [1]. Among the various AM techniques, Fused Filament Fabrication (FFF) is one of the most widely used due to its simplicity, accessibility, and cost-effectiveness. Unlike alternative AM methods that often require costly equipment, specialized training, or controlled environments, FFF relies on filament feedstocks that are heated, melted, and extruded through a simple deposition tool. 3D printing is achieved by dispensing the semisolid material while controlling the relative motion between the deposition tool and the print bed. This inherent simplicity has facilitated the widespread availability of both industrial and consumer-grade FFF printers at affordable prices [2].

Various general-purpose polymers are available as filament feedstock for FFF, including polylactic acid (PLA), acrylonitrile-butadiene-styrene (ABS), polyethylene terephthalate glycol-modified (PETG), thermoplastic polyurethane (TPU), and polyamide (PA). High-performance engineering plastics like polyphenylene sulfide (PPS), polysulfone (PSU), polyetherimide (PEI), and, more recently, polyether ether ketone (PEEK) can also be printed [3]. However, their processing requires high-temperature deposition tools and temperature-controlled build chambers, making these materials more suitable for industrial equipment than for consumer-grade 3D printers [4].

As a high-performance thermoplastic, PEEK has become increasingly sought after by industries that require durable and lightweight materials and is often used as a substitute for aluminum and titanium, especially in the medical sector. PEEK is extremely resistant to wear, hydrolysis, radiation, and most chemicals and can maintain high strength and dimensional stability even at elevated temperatures of 250 °C for long periods of time [5,6]. Due to its biocompatibility and ability to withstand repeated sterilization, PEEK can be used to fabricate orthopedic implants, spinal fusion devices, dental implants, and surgical instruments [7,8]. In addition, since its mechanical properties closely resemble human bone, PEEK can be an ideal choice for medical implants [9].

With a crystallization temperature from the melt of approximately 290 °C [6], PEEK is considered a slow-crystallizing polymer when compared to non-aromatic polymers, typically achieving crystallinity levels below 50% [10]. Crystallization is usually indicated by a visible color change from transparent dark brown to opaque beige [11]. Although higher crystallinity enhances the mechanical performance of the final part, excessive crystallization during printing may lead to shrinkage and warping. Moreover, it has been reported that crystalline domains can hinder interlayer bonding, thereby reducing mechanical strength [12]. In this context, the high cooling rates inherent to 3D printing make PEEK grades with limited crystallization under such conditions preferable. Additional crystallization can then be promoted through post-printing annealing, during which the material should be heated above 180 °C [6].

The mechanical properties of 3D-printed PEEK and their dependence on process parameters have been studied mostly under static conditions. Zanjanijam et al. [13] provide a broad overview of PEEK’s tensile and compressive behavior, including typical values of strength, modulus, and elongation, as well as a summary of printing parameters used in the literature. Moby et al. [14] additionally review studies exploring the suitability of PEEK for dental restorations.

Regarding tensile behavior, Yang et al. [15] examined how chamber and nozzle temperature, as well as post-printing heat treatments affect crystallinity and mechanical performance. Increasing the chamber temperature from 25 °C to 200 °C raised crystallinity from 16.9% to 31.16% and improved tensile strength up to 84 MPa. Higher nozzle temperatures enhanced interlayer bonding, while annealing produced the highest crystallinity (37.6%), consistent with other studies [16,17,18,19,20,21] showing that annealing increases strength but reduces ductility. Rendas et al. [16] found optimal tensile strength (86.7 MPa) at 495 °C nozzle temperature, 130 °C chamber temperature, 0.1 mm layer height, and an extruder multiplier of 1.1. Using micro-computed tomography (m-CT), they correlated higher tensile strength to lower void content and identified ±45° infill as superior due to a “scissoring” deformation mechanism. Annealing at 300 °C for 4 h further increased tensile strength by up to 10% and modulus by up to 30%. Other works [17,19,20] report similar reductions in void area after annealing, although Basgul et al. [22] found no significant porosity change and even a decrease in strength.

The number of works exploring the influence of 3D printing parameters in final compressive properties is smaller. Rendas et al. [23] showed concentric infill yields 45% fewer voids than rectilinear patterns, resulting in a higher effective cross-section and thus improving compressive properties. Basgul et al. [24] demonstrated that FFF PEEK lumbar cages achieve 63–71% of the compressive and shear strength of machined cages and 92% of their torsional strength. Despite lower mechanical properties compared to machined or injection-molded PEEK, the compressive strength of the printed cages (~8000 N) exceeds typical lumbar spine loads (1000–3000 N) [25,26] and remains within the required range for interbody fusion implants [27], supporting the viability of additively manufactured PEEK in medical applications.

Despite its potential applications where cyclical loading regimes are typically expected, fatigue characterization of 3D-printed PEEK parts is still incipient. Rendas et al. [28] reported high-cycle tensile fatigue behavior using ASTM D638 specimens and found a fatigue strength of 65 MPa (≈75% of tensile strength), consistent with bulk PEEK. Greco et al. [29] demonstrated that infill pattern has a stronger influence on fatigue life than layer height, with rectilinear infill achieving the best performance and infinite life at 70% of tensile strength. In the biomedical field, Surendrasingh et al. [30] evaluated FFF PEEK dental implants under ISO 14801 loading and found satisfactory fatigue performance, though the study focused on implant-level behavior rather than fundamental material characterization.

Since 3D printing of PEEK is particularly attractive for medical applications, where patient-specific implants and the material’s biocompatibility and bone-like properties offer clear advantages, characterizing its fatigue performance under compression is essential. To the best of the authors’ knowledge, no S–N curves for the compressive fatigue behavior of 3D-printed PEEK are currently available. Consequently, the influence of annealing on its compressive fatigue performance also remains unknown, underscoring the need for further investigation to ensure the material’s suitability for load-bearing medical applications subject to cyclic loading.

The objective of this study is to address existing gaps in the literature by investigating the static tensile and compressive behavior, as well as the compressive fatigue performance, of 3D-printed PEEK. The effects of infill raster angle and annealing on these mechanical properties are evaluated to assess the material’s suitability for structural and biomedical applications.

## 2. Materials and Methods

### 2.1. 3D Printing and Annealing

All specimens were manufactured on a 3DGence Industry F420 printer (3DGence, Przyszowice, Poland) using 1.75 mm 3DGence PEEK Natural filament [31]. Two layers of Dimafix glue (I3D Digital Media S.L., Almería, Spain) were applied to the glass build plate, and a raft made from ESM-10 support material, deposited through the secondary nozzle, was used to ensure proper adhesion. Prior to printing, the PEEK filament was dried at 60 °C for at least 24 h, and the support filament was stored in a low-humidity cabinet at room temperature. The printing parameters listed in Table 1 were defined in 3DGence Slicer 4.0 (3DGence, Przyszowice, Poland). Unlisted settings were kept at the default values recommended for PEEK and ESM-10 (3DGence, Przyszowice, Poland).

Thirteen ISO 527 type 1BA tensile specimens were sequentially printed in a single batch, following the YX orientation from ASTM F2971 [32]. Seven of these tensile specimens were annealed, and six were tested as printed. For the static compression tests, nine cylindrical specimens defined according to the ASTM D695 [33] were individually printed: five with a ±45° raster that were kept as printed and four with a ±45° raster that were subsequently subject to annealing. For the fatigue tests, seventeen cylindrical specimens with a ±45° raster angle were also printed individually, comprising six to be annealed and eleven to be kept as printed. The ±45° raster orientation was chosen because it produced specimens with fewer visible 3D printing defects and potentially led to superior strength and interlayer adhesion [16]. After manufacturing, the specimens were sealed inside zip-lock bags with silica packets and stored in a low-humidity storage cabinet.

Annealing was performed using a high-precision SLW 53 drying oven (POL-EKO, Wodzisław Śląski, Poland). The tensile specimens were placed between 5 mm aluminum plates to prevent warping. Heating was done slowly (2 °C/min) to ensure uniform heating and reduce internal stresses. The material was kept at 155 °C for 1 h to reduce residual stresses, followed by 5 h at 270 °C. After that the oven was turned off to allow for slow cooling.

### 2.2. Differential Scanning Calorimetry (DSC)

Differential scanning calorimetry (DSC) analysis was carried out using a TA Instruments DSC 250 (TA Instruments, Waters Corporation, Milford, MA, USA) in three stages to evaluate the degree of crystallinity of the printed PEEK tensile specimens, with and without annealing. First, a heating cycle of 10 °C/min from room temperature to 420 °C was conducted, followed by cooling to room temperature again at 10 °C/min. The second heating cycle was performed in the same conditions as the first one. The test was done with a nitrogen atmosphere. DSC samples were taken from the middle portion of the specimen on its gauge length, in the as-printed condition. Crystallinity percentage $X_{C}$ was calculated according to Equation (1).

(1) $$X_{C}=\frac{{∆H}_{m}-{∆H}_{cc}}{{∆H}_{m}^{0}}\times 100$$

where ${∆H}_{m}\;$(J/g) represents the enthalpy of melting, ${∆H}_{cc}$ (J/g) is the enthalpy of cold crystallization, and ${∆H}_{m}^{0}\;$(130 J/g) is the enthalpy of melting for 100% crystalline PEEK [6].

### 2.3. Static Tensile and Compression

Tensile testing was conducted using an Instron 5566 dual-column universal testing machine (Instron Corporation, Norwood, MA, USA), which recorded the displacement between the grips in millimeters (mm) and the corresponding force in Newtons (N). Mechanical grips were used to secure the samples, with the initial distance fixed at 75 mm. A crosshead speed of 0.8 mm/min was applied, corresponding to the 1% strain per minute specified in the ISO 527 standard [34]. The elastic modulus was determined from the slope of the initial linear region of the stress–strain curves, and the ultimate tensile strength (UTS) was obtained as the maximum stress reached on the curve prior to fracture.

Compression testing was conducted using an Instron 3369 universal testing machine (Instron Corporation, Norwood, MA, USA). Testing was done under displacement control at 1.3 mm/min according to the ASTM D695 standard [35], and a preforce of 100 N was applied before starting the test. The test stopped either upon failure or when displacement reached 20% of specimen height, which corresponds to 5 mm. All testing was performed at around room temperature of 23 °C. The “C” before the specimen number identifies it as a compression specimen.

### 2.4. Micro-Computed Tomography (Micro-CT)

Micro-CT analysis was performed on a single tensile test specimen using a Bruker Skyscan 2214 (Bruker Corporation, Billerica, MA, USA). A voxel size of 16 µm was used. The specimen was submerged in liquid nitrogen and subsequently broken in half using pliers. One half of the fractured specimen was then subjected to the same annealing cycle applied to the remaining batch, while the other half remained in the as-printed condition. The scan was conducted in the narrow section of the specimen with some distance maintained from the radius transition and the fracture surface to ensure a constant cross-section. The selected specimen had not been previously tested and was manufactured in the same conditions as the rest of the batch used for static testing.

### 2.5. Fatigue Compression Testing

**Figure 1:**
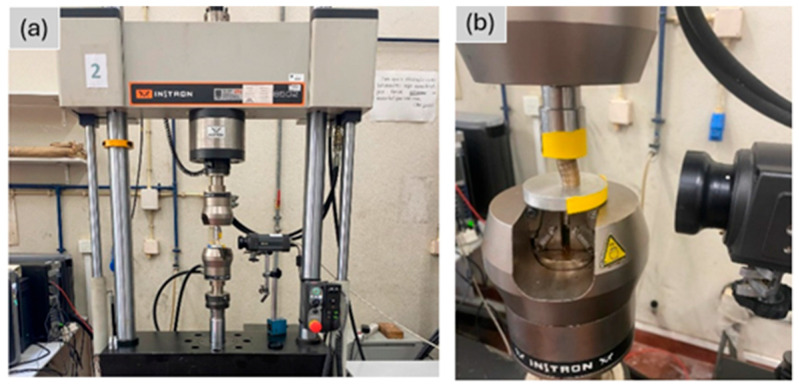
(**a**) Photograph of the Instron 8502 Servohydraulic Universal Testing Machine used for fatigue testing; (**b**) photograph of the thermal camera setup.

Cyclic testing was performed using an Instron 8502 Servohydraulic Universal Testing Machine (Instron Corporation, Norwood, MA, USA), as depicted in Figure 1a. The temperature of specimens was monitored using a FLIR A300 thermal imaging camera (Teledyne FLIR LLC., Wilsonville, OR, USA), shown in Figure 1b, that recorded their surface temperature inside a predefined area as a function of the time elapsed.

Prior to testing, some samples were spray-painted black to increase the emissivity of the surface and ensure a more accurate temperature measurement; however, adjusting the emissivity on the camera settings also proved sufficient for adequate temperature readings. Specimens were also ground down by 0.2 mm to make sure the load was better aligned and to avoid slipping during testing. A compression–compression cyclic sinusoidal loading regime was employed using a stress ratio of R = 0.1. The tests were load-controlled with maximum force values ranging from 70% to 95% of the maximum compressive force obtained in the static tests (this percentage applied was designated as Stress Level). Frequency values of 5 Hz and 2.5 Hz were used, and a preforce of 100 N was applied prior to starting the test. At first the tests were stopped if specimen height was reduced by 20%, but this was later changed to stop either when specimen height was reduced by 10%, which corresponds to 2.5 mm, or when 1 million cycles were reached. All tests were conducted at room temperature of around 23–25 °C.

The resulting test data was used to construct the Wöhler curve, illustrating the relationship between stress amplitude ($\sigma_{amp}$) and the number of cycles to failure ($N_{f}$). A curve was fitted to the data using Equation (2), where *A* and *b* are constants.

(2) $$\sigma_{amp}=A{N_{f}}^{b}$$

## 3. Results and Discussion

### 3.1. PEEK Thermal Properties

**Figure 2:**
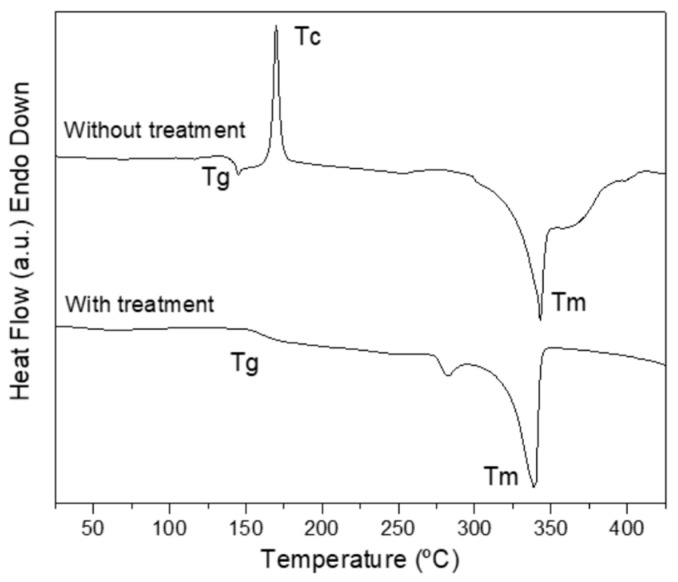
DSC curves of the first heating cycle performed on annealed and as-printed PEEK samples.

Figure 2 shows the DSC curves from the first heating cycle, reflecting the material state after 3D printing and after annealing. As indicated by the color of the printed parts, the as-printed sample exhibits a cold-crystallization peak at ~175 °C, confirming that the PEEK grade used cannot fully crystallize under typical printing conditions [6]. A broad exothermic peak near 280 °C further suggests that additional crystallization, associated with the formation of more perfect crystallites, can occur upon heating. In contrast, the annealed sample shows no exothermic peaks, indicating that the treatment successfully induced the maximum crystallinity attainable under these conditions.

The double endothermic region observed in the annealed specimen reflects the presence of distinct crystal populations: smaller, less perfect crystals melting at lower temperatures, followed by more perfect crystallites formed at higher annealing temperatures [36]. After melting, the annealed sample displays a stable heat-flow signal, whereas the unannealed sample shows noticeable fluctuations. This behavior is attributed to the greater thermal stability and microstructural homogeneity of the annealed material, while the as-printed specimen continues to undergo structural reorganization during heating.

**Figure 3:**
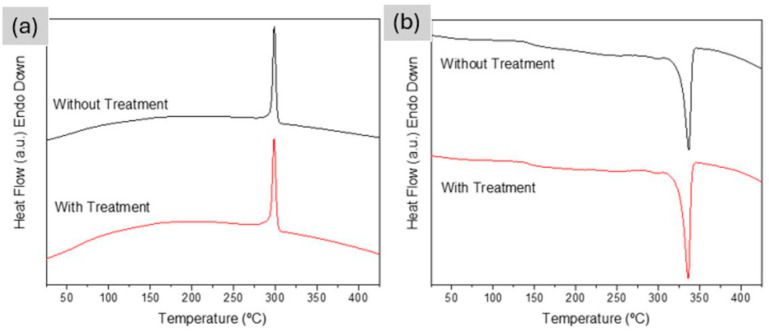
DSC curves of as-printed and annealed PEEK samples: (**a**) cooling cycle, (**b**) second heating cycle.

The cooling curve in Figure 3a shows a single crystallization event from the melt at approximately 300 °C for both the as-printed and annealed samples. In the second heating cycle, shown in Figure 3b, which reflects the material behavior after erasing its prior thermal history, a single, well-defined melting peak appears around 330 °C, with no significant difference between the two conditions. These results confirm that the annealing treatment effectively increased crystallinity from 34.4% in the as-printed state to 41.4% after annealing, a 7% increase.

### 3.2. Static Tensile and Compressive Behavior

**Figure 4:**
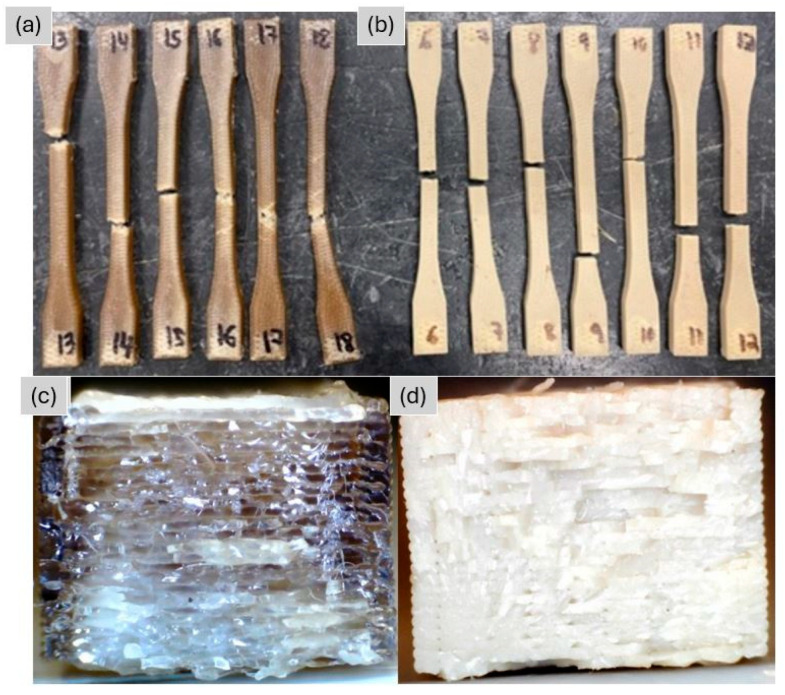
Photograph of fractured tensile specimens, tested in the (**a**) as-printed and (**b**) annealed conditions. Cross-section of specimens (**c**) 14 and (**d**) 8.

The fractured tensile specimens are shown in Figure 4a,b. A clear distinction in fracture behavior is evident: unannealed specimens display irregular fracture surfaces with pronounced filament pullout, depicted in Figure 4c, whereas annealed specimens exhibit straighter, more brittle fracture planes, depicted in Figure 4d. Specimens 13 and 9 were excluded from analysis because failure occurred within the grip transition region.

**Figure 5:**
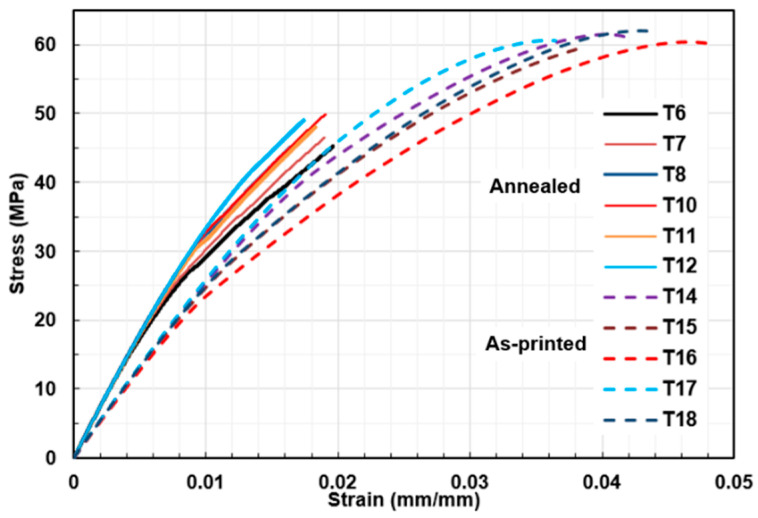
Strain–stress diagram of ISO tensile specimens with (solid line) and without annealing (dashed line), both with ±45° raster orientation.

The tensile stress–strain curves in Figure 5 reveal that, contrary to trends reported in the literature, annealing did not improve tensile strength. The as-printed specimens achieved a higher average UTS (60.77 ± 0.9 MPa) compared to the annealed ones (47.31 ± 1.84 MPa). As expected, Young’s modulus increased after annealing, rising from 2.51 ± 0.03 GPa to 3.51 ± 0.03 GPa. Strain at break also differed significantly, decreasing by almost half from 0.040 in as-printed specimens to approximately 0.018 in annealed ones.

Although crystallinity increased after annealing, the unexpected reduction in UTS is likely explained by the intensification of pore severity caused by thermally induced shrinkage. This interpretation is supported by the measured dimensional contraction of the annealed specimens (0.7–1.8% in length and 0.4–1.6% in width), which aligns with known post-annealing behavior in thermoplastics [37]. This hypothesis was subsequently evaluated using the compression results and M-CT analysis.

**Figure 6:**
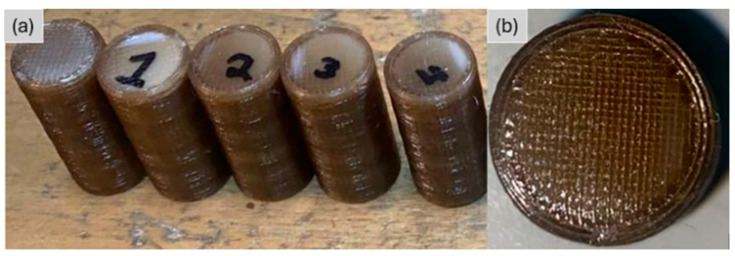
(**a**) Photograph of as-printed PEEK compression specimens (**b**) close-up view of their cross-section, showing the ±45° raster orientation.

**Figure 7:**
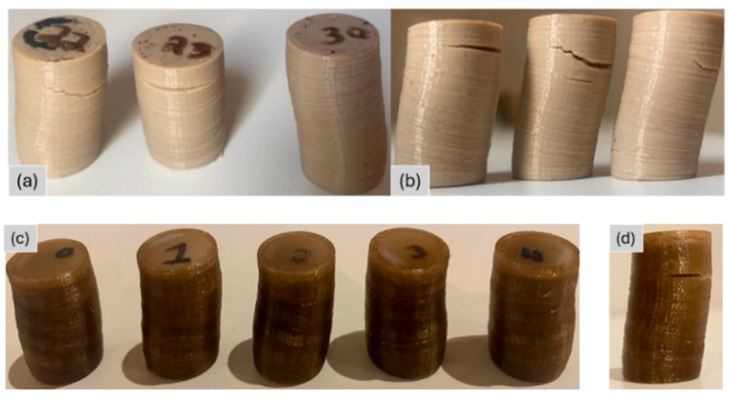
Photographs of as-printed and annealed PEEK specimens after static compression testing: (**a**) annealed; (**b**) annealed, side view; (**c**) as-printed; (**d**) as-printed, side view.

The cylindrical compression specimens printed with ±45° raster orientation are presented in Figure 6 and Figure 7. Opaque regions on their top surfaces signal localized crystallization driven by heat accumulation during FFF. The areas near the corners, which cool more slowly, show color changes associated with higher crystallinity. These patterns arise from the layer-by-layer deposition process, in which repeated passes of the nozzle locally retain heat and promote crystal formation in semi-crystalline polymers such as PEEK [6,10].

The resulting anisotropic structure, combined with printing defects such as imperfect interlayer bonding and internal voids, promotes localized failure in weaker regions. Under loading, printed layers progressively collapse onto one another, leading to specimen buckling, as shown in Figure 7b,d. The failure mode of the annealed specimens was similar to that of the as-printed ones. Buckling was observed, and delamination occurred along the sides of the specimens in between printed layers, indicating anisotropic material behavior, as shown in Figure 7. However, the annealed specimens exhibited improved interlayer adhesion, as delamination did not cleanly occur between individual layers. Instead, fracture surfaces extended across multiple layers, indicating stronger bonding between them.

**Figure 8:**
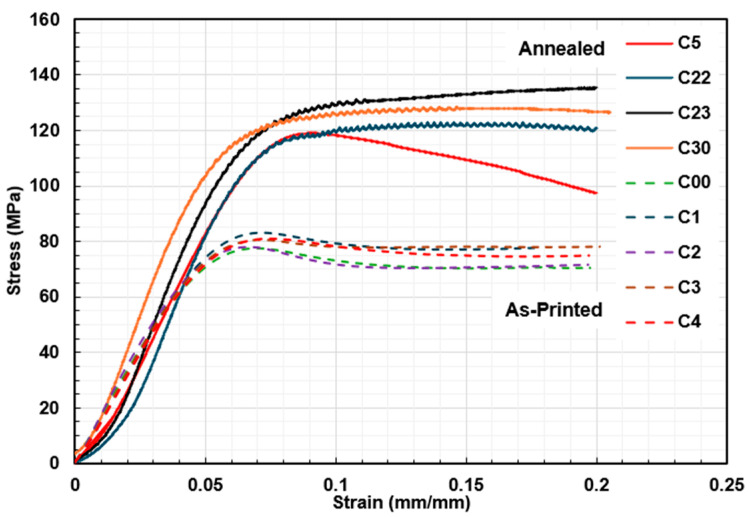
Strain–stress diagram of compression specimens with (solid line) and without annealing (dashed line), both with ±45° raster orientation.

The stress–strain curves for the as-printed and annealed compression specimens are presented in Figure 8. The material exhibits a typical ductile behavior with a stress plateau after the elastic region.

Despite the fluctuations observed in the annealed specimens, which are attributed to instabilities in the testing machine’s load control and resulted in a low-amplitude sinusoidal response, annealing clearly enhanced the compressive properties. The maximum compressive strength increased from an average of 80.12 ± 2.3 MPa in the as-printed condition to 126.68 ± 7.3 MPa after annealing, corresponding to an improvement of 58.12%. A similar trend was observed for the compressive modulus, which increased by 39.02% from 1.64 ± 0.05 GPa to 2.28 ± 0.24 GPa. These values are consistent with previous reports of compressive strengths of approximately 120 MPa [17] and 147 MPa [18] for annealed specimens and are comparable to those reported for bulk PEEK.

These results provide clear evidence that the annealing process effectively enhances mechanical properties through increased crystallinity, particularly in loading conditions where failure is not governed by stress concentrations under tensile loading arising from larger voids.

### 3.3. Mesostructural Analysis

**Figure 9:**
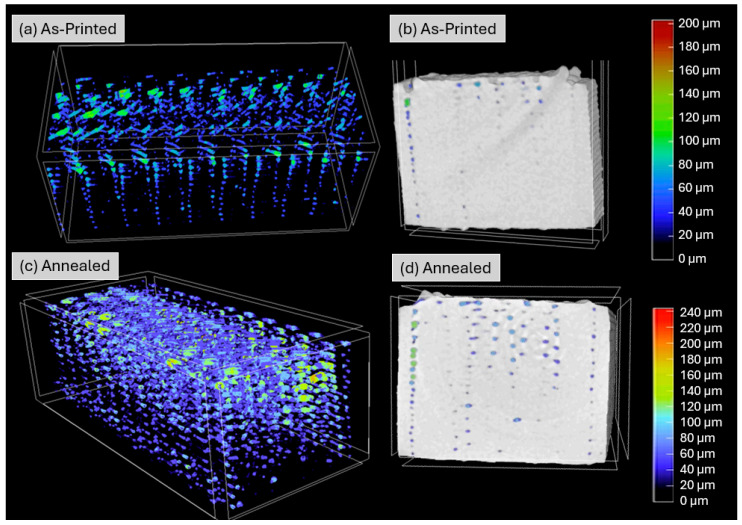
Micro-CT 3D rendering of the internal porosity distribution in the (**a**) as-printed and (**c**) annealed halves of the PEEK tensile specimens. Micro-CT cross-sectional image in the (**b**) as-printed and (**d**) annealed states.

Figure 9 provides a visual representation of the internal porosity of the printed PEEK tensile specimen. Compared to the as-printed condition shown in Figure 9a,b, the annealed sample shown in Figure 9c,d exhibits noticeably larger pores, predominantly aligned along a ±45° orientation throughout the part. This alignment is consistent with the deposition paths, as voids typically form parallel to the printed lines and thus reflect the raster angle used in the FFF process. In addition, the voids exhibit a vertically repetitive pattern, which arises from the layer-by-layer deposition inherent to FFF and the resulting interlayer gaps between successive printed layers.

It is also evident that the upper side of the cross-section has more pore presence than the lower side, which is more easily seen in Figure 9b,d. This upper side corresponds to the layer that was first printed and was in contact with the raft. The large thermal difference in the first layer reduced the adhesion of subsequent layers and introduced voids, showing that the first layer is crucial for the correct 3D printing of PEEK.

**Figure 10:**
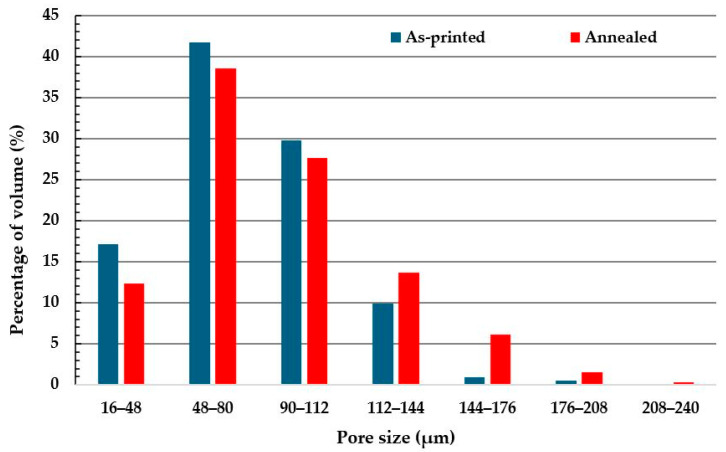
Pore size distribution of as-printed (**blue**) and annealed (**red**) halves of 3D-printed PEEK tensile specimens.

Figure 10 presents the pore size distribution for both halves of the same specimen. The shift in pore size is evident: in the as-printed half, 88.66% of the pores fall within the 16–112 μm range, whereas in the annealed half, only 78.53% remain in that range. Consequently, pore size in the 112–240 μm range increased from 11.34% to 21.47% with annealing. Since these two halves originated from the same specimen, this shift in distribution confirms that pore size increased with annealing from an average pore size of 75.96 μm to 85.69 μm.

In material-extrusion AM, voids originate from incomplete fusion between adjacent rasters and limited interlayer adhesion. The tensile results obtained after annealing can be interpreted considering the interplay between crystallization and the evolution of these pre-existing defects. DSC analysis showed that annealing increased the crystallinity of the material from 34.4% to 41.4%. While higher crystallinity typically increases stiffness, it is also associated with volumetric contraction during crystal formation. In a printed mesostructure already containing interlayer voids, this contraction can enlarge existing pores and promote pore coalescence [38]. This interpretation is supported by the micro-CT results, which revealed an increase in porosity from 0.5% to 2.15% after annealing, despite previous reports suggesting that annealing may reduce porosity in additively manufactured PEEK [19,20]. Given the well-established sensitivity of tensile strength to void content in 3D-printed PEEK [16,19,20,21], the enlargement of pores acting as stress concentrators provides a plausible explanation for the observed reduction in tensile strength.

The microstructural evolution during annealing can also be interpreted in light of the competition between crystallization and interlayer molecular diffusion. Domenech et al. [39] demonstrated for polypropylene that rapid crystallization can restrict molecular interdiffusion and entanglement across deposited interfaces, limiting neck growth and interlayer strength. Although their work focused on early post-deposition solidification in a different polymer system, the underlying mechanism is similar. During annealing, molecular mobility increases, enabling further crystallization within the bulk; however, because the material remains below its melting temperature, the geometry of the deposited beads and their contact area remain essentially fixed. Under these conditions, crystallization and the associated shrinkage can proceed without significant improvement in interlayer bonding, favoring the amplification of pre-existing defects rather than their healing.

The compression results provide further support for this interpretation. While tensile strength decreased after annealing, compressive strength increased. This behavior is consistent with the increase in crystallinity, which enhances stiffness and load-bearing capacity under compressive loading. If the dominant factor controlling mechanical performance were solely the change in crystallinity or potential material degradation, similar reductions would be expected in both tensile and compressive responses. Instead, the divergent trends indicate that tensile performance is primarily governed by the growth and coalescence of pores acting as stress concentrators, whereas compressive behavior remains largely controlled by the bulk stiffness of the crystalline matrix. This interpretation aligns with the micro-CT observations and the known sensitivity of tensile properties to porosity in additively manufactured PEEK.

### 3.4. Compression Fatigue Results

The results of the PEEK compression fatigue tests are presented in Table 2, divided by specimen condition (as-printed or annealed), applied loading frequency, and stress amplitude. The results are organized in descending order of stress level, where stress level refers to a percentage of the maximum applied compressive stress, based on prior static testing.

**Figure 11:**
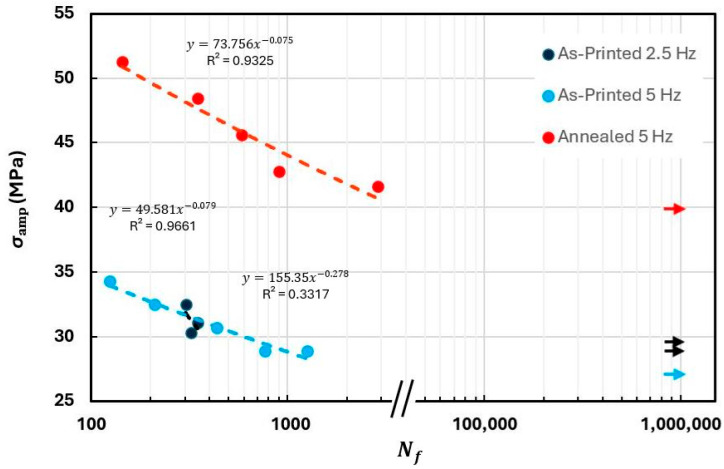
S-N plot of the experimental data of PEEK compression specimens. Arrows indicate run-out conditions at each frequency and follow the same color scheme as the data points.

Using the reported values, S-N curves were constructed as shown in Figure 11. The Basquin’s model fit variables (*A* and *b*) and the correlation coefficient are also displayed in the figure for each curve.

The tested specimens exhibited one of two behaviors: either they failed rapidly (before 10^4^ cycles) due to thermal effects, or they achieved infinite life, meaning that they reached one million cycles without meeting the test stop criterion. Notably, no specimen failure was observed beyond 2871 cycles.

The results confirm that at higher stress amplitudes, specimens endure fewer cycles before failure, while lower stress levels eventually approach the material’s fatigue limit. On both frequencies 2.5 Hz and 5 Hz, this trend was verified.

The annealed specimens demonstrated improved fatigue performance, enduring a higher number of cycles at greater applied stress levels compared to the unannealed specimens.

The fatigue limit for 3D-printed PEEK under compression ranged from 75% to 82% (60.1–65.7 MPa) of the material’s maximum static compressive stress in the as-printed condition and was approximately 70% (88.61 MPa) in the annealed state. Although the fatigue limit represented a smaller percentage of the static strength in the annealed state, the actual stress amplitude was 40.9% higher in annealed specimens, demonstrating its improved fatigue life. Although no previous studies have specifically investigated the compressive fatigue behavior of 3D-printed PEEK, existing research on tensile fatigue typically reports fatigue limits in the range of 70% to 75% of the ultimate tensile strength [40]. The compression fatigue limit obtained in the present study aligns with, and in some cases surpasses, the tensile fatigue values of other 3D-printed PEEK studies. These findings support the potential suitability of 3D-printed PEEK for fatigue applications under compression loading.

### 3.5. Thermal Analysis Under Cyclic Loading

**Figure 12:**
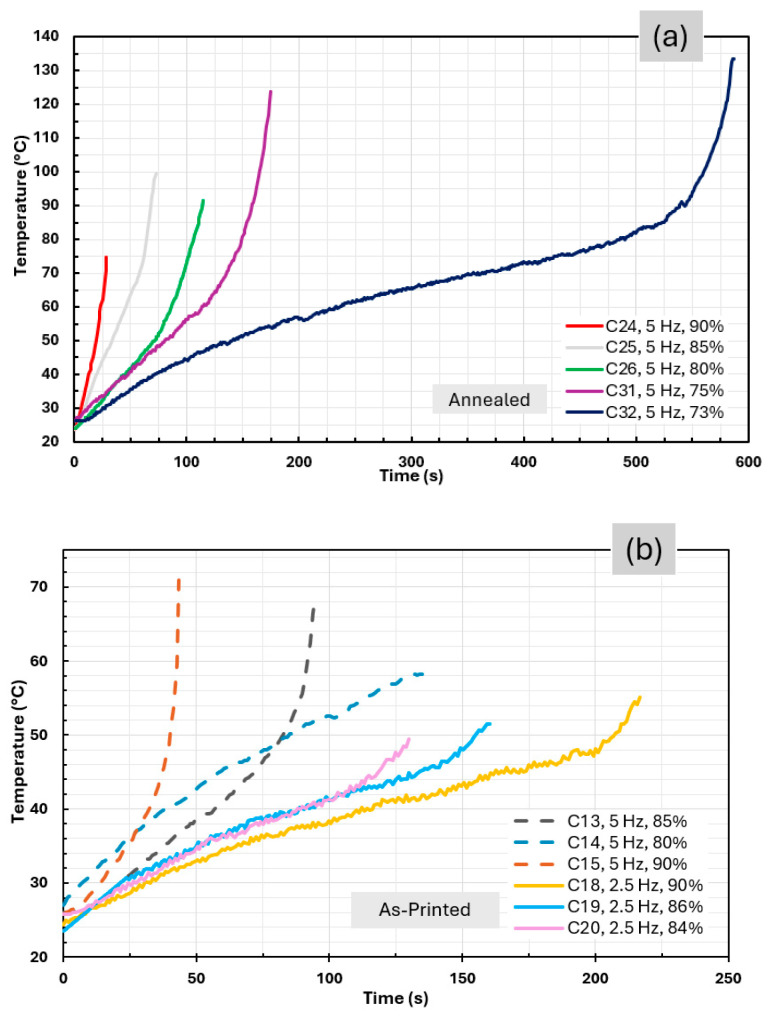

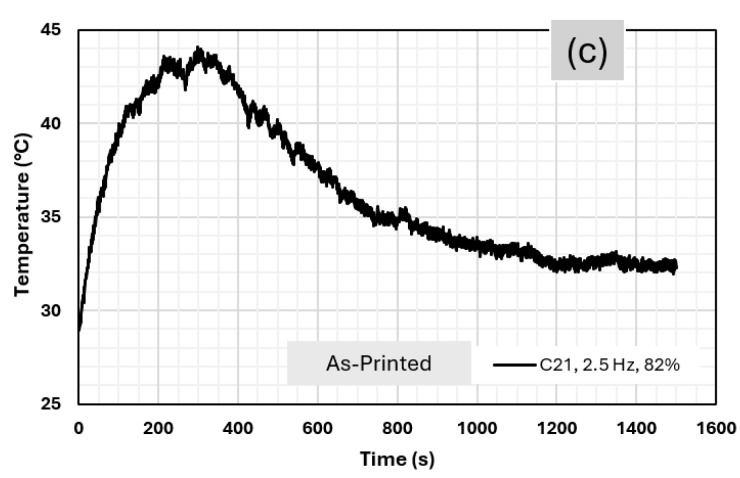
Temperature evolution plot of: (**a**) annealed specimens; (**b**) as-printed specimens; (**c**) infinite-life specimen C21.

Overall, two thermal behaviors were observed: (i) a continuous temperature increase leading to thermal failure (Figure 12a,b), and (ii) an initial temperature rise followed by stabilization and reduction, indicating the attainment of thermal equilibrium (Figure 12c).

The first behavior results from hysteretic heating during cyclic deformation, where heat generation exceeds dissipation, causing thermal softening and early failure, particularly at higher stress amplitudes or frequencies. The onset of accelerated heating occurred at higher temperatures in annealed specimens (~80 °C) than in unannealed ones (~46 °C), demonstrating improved thermal stability due to annealing. In contrast, specimens reaching thermal equilibrium transitioned to a mechanically dominated fatigue regime and achieved infinite life [41].

**Figure 13:**
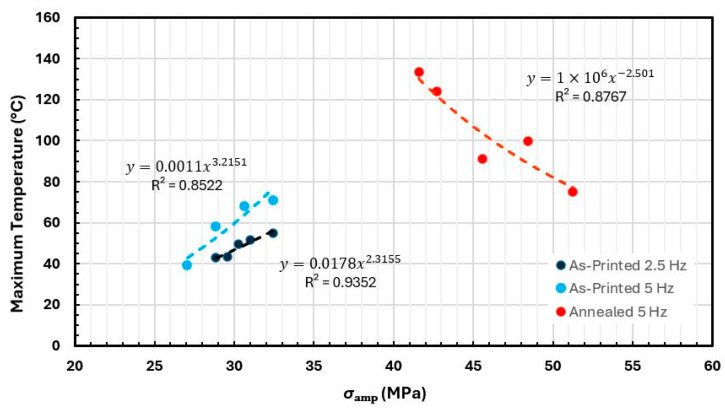
Plot of stress amplitude vs. maximum temperature for as-printed and annealed specimens.

These findings highlight the critical role of temperature control in fatigue testing of 3D-printed PEEK, as uncontrolled self-heating can lead to premature failure and misinterpretation of fatigue performance. Figure 13 shows the dependence of maximum temperature on stress level during cyclic loading for as-printed and annealed specimens.

As-printed specimens exhibit increasing maximum temperatures with increasing stress, consistent with enhanced hysteretic heating due to greater deformation, in agreement with the literature [41]. Higher loading frequencies further amplified temperature rise at a given stress level, reflecting the increased rate of energy dissipation. Specimens that reached infinite life displayed the lowest peak temperatures, highlighting the strong coupling between temperature and fatigue behavior in PEEK. In contrast, annealed specimens showed the opposite trend, with higher stresses leading to lower maximum temperatures. This behavior is attributed to increased stiffness and brittleness after annealing, which reduces deformation and hysteretic heating, promoting more brittle failure with limited thermal accumulation. At lower stress levels, longer fatigue lives allowed greater heat buildup. Overall, annealed specimens demonstrated improved thermal stability under cyclic loading.

### 3.6. Fatigue Failure Analysis

**Figure 14:**
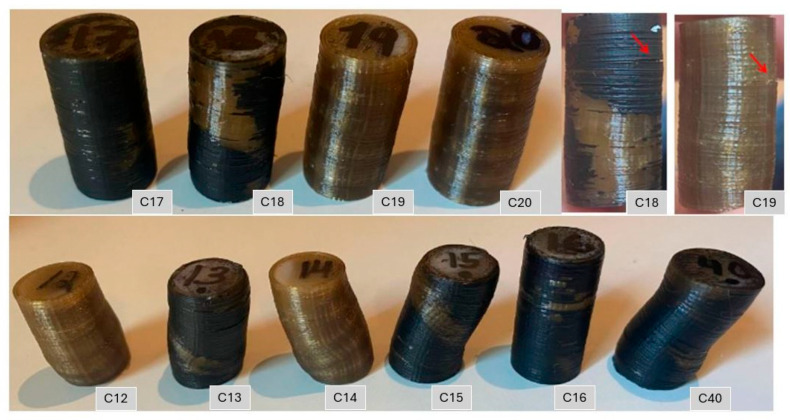
Photograph of as-printed PEEK fatigue compression specimens C17–C20 (2.5 Hz) and C12–C16 and C40 (5 Hz). Signs of delamination are indicated with red arrows.

The as-printed PEEK specimens tested at a frequency of 2.5 and 5 Hz (shown in Figure 14) exhibited similar failure mechanisms, dominated by anisotropic behavior characterized by delamination (red arrows in Figure 14), and buckling rather than classical barreling. This response is consistent with the static compression results and highlights the influence of weak interlayer adhesion in 3D-printed PEEK.

Most specimens failed prematurely, while those tested at lower stress amplitude (C16 and C17, 80%) achieved infinite life and showed minimal deformation, remaining within the fatigue endurance limit. The lower apparent deformation at 2.5 Hz was primarily the consequence of a stricter test stop criterion (10% height reduction instead of 20%), rather than an intrinsic improvement in fatigue behavior.

**Figure 15:**
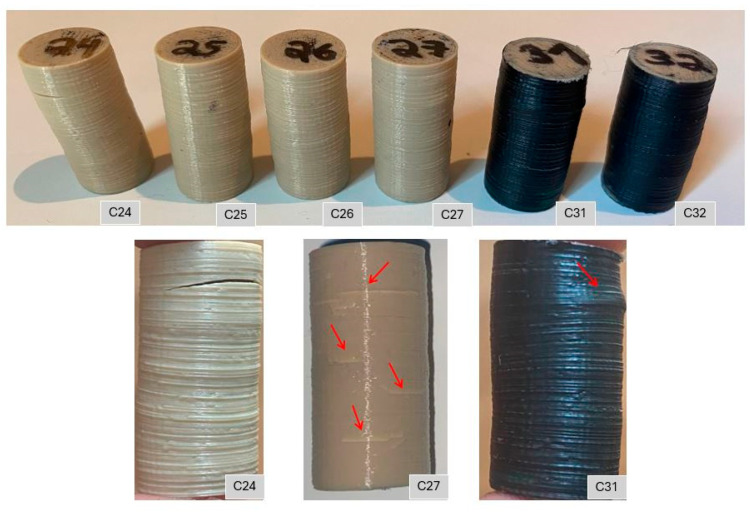
Photograph of annealed PEEK fatigue compression specimens C24–C27 and C31–C32 (5 Hz). Red arrows indicate cracks and layer collapse for specimens C27 and C31 respectively.

In contrast, the annealed specimens tested at 5 Hz, presented in Figure 15, showed reduced deformation and a more brittle failure mode, consistent with increased crystallinity. Although delamination remained the dominant failure mechanism, buckling was significantly less pronounced, indicating reduced anisotropy and improved interlayer cohesion. Specimen C27 reached infinite life with no visible delamination but with cracks starting to form (red arrows in Figure 15, specimen C27), while others exhibited either multilayer delamination (e.g., C24) or localized layer collapse (red arrow in Figure 15, specimen C31). The observation that specimen C26, tested at a higher stress level than C31, showed only barreling highlights the inherent variability of additively manufactured parts, likely associated with localized defects and porosity arising from printing inconsistencies.

## 4. Conclusions

This study investigated the static and cyclic mechanical behavior of 3D-printed PEEK, with particular emphasis on the influence of annealing and with a focus on compressive loading conditions relevant to biomedical applications. Tensile static performance was negatively affected by annealing due to an increase in pore size, highlighting the need for further process optimization to improve interlayer bonding and defect control. The results demonstrate that annealing significantly enhances compressive strength, stiffness, and fatigue performance, enabling the material to withstand cyclic compressive stresses up to ~70% of its static strength for 10^6^ cycles. A key contribution is the experimental characterization of compressive fatigue behavior of 3D-printed PEEK, addressing a notable gap in the existing literature.

When compared with typical in-service conditions of intervertebral spinal cages [26,27], the mechanical performance of annealed 3D-printed PEEK largely exceeds the compressive stress levels encountered in vivo, indicating clear potential for load-bearing spinal applications. While these findings support the feasibility of using additively manufactured PEEK for patient-specific implants, the conclusions remain preliminary since a comprehensive evaluation under simulated physiological conditions would require a dedicated fatigue-testing scheme, which is beyond the scope of the present study.

Overall, the combination of biocompatibility, bone-like mechanical properties, and adequate compressive fatigue resistance positions 3D-printed PEEK as a promising candidate for customized biomedical implants. Future work should focus on porosity mitigation, long-term durability, and application-specific validation to fully establish its clinical viability.

## Figures and Tables

**Table 1:** 3D-printing parameters used to fabricate PEEK specimens.

Layer height	0.15 mm	Printing speed	50 mm/s (tensile) 25 mm/s (compression and fatigue)
Perimeter walls count	2	Perimeter walls speed	40 mm/s
Infill pattern	±45°	Initial layer speed	30 mm/s (tensile) 15 mm/s (compression and fatigue)
Infill density	100%	Number of slower layers	1
Printing temperature	420 °C	Support adhesion	Raft
Build plate temperature	130 °C	Raft extra margin	5
Build volume temperature	110 °C	Support printing temperature	260 °C
Cooling fan	On	Support layers count	3
Extrusion flow	90%	Support infill pattern	±45°

**Table 2:** Results of PEEK fatigue compression tests.

	Specimen	Stress Level (%)	σ_amp_ (MPa)	Cycles to Failure	Max. Specimen Temperature (°C)
As-printed 2.5 Hz	C18	90	32.4	302	55.1
	C19	86	31.0	348	51.5
	C20	84	30.3	320	49.4
	C21	82	29.6	Run-out	44
	C17	80	28.8	Run-out	42.8
As-printed 5 Hz	C40	95	34.3	124	-
	C15	90	32.4	210	70.9
	C13	85	30.6	434	68.1
	C14	80	28.8	763	58.3
	C12	80	28.8	1251	-
	C16	75	27.0	Run-out	39.2
Annealed 5 Hz	C24	90	51.3	143	75
	C25	85	48.4	346	99.6
	C26	80	45.6	583	91.3
	C31	75	42.7	897	123.9
	C32	73	41.6	2871	133.6
	C27	70	39.9	Run-out	73.8

## Data Availability

The data presented in this study are available on request from the corresponding author.
